# *rac*-Ethyl 3-(3-bromo-2-thien­yl)-2-oxo-6-(4-propoxyphen­yl)cyclo­hex-3-ene-1-carboxyl­ate

**DOI:** 10.1107/S1600536808032650

**Published:** 2008-10-22

**Authors:** Andreas Fischer, M. T. Swamy, B. Narayana, H. S. Yathirajan

**Affiliations:** a Inorganic Chemistry, School of Chemical Science and Engineering, Royal Institute of Technology (KTH), 100 44 Stockholm, Sweden; b Department of Studies in Chemistry, University of Mysore, Manasagangotri, Mysore 570 006, India; c Department of Studies in Chemistry, Mangalore University, Mangalagangotri 574 199, India

## Abstract

The racemic title compound, C_22_H_23_BrO_4_S, crystallizes with two mol­ecules in the asymmetric unit. The dihedral angles between the thio­phene and phenyl rings are 71.64 (17) and 73.41 (17)°.

## Related literature

For general background, see: House (1972 ▶); Tabba *et al.* (1995 ▶); Dimmock *et al.* (1999 ▶); Dhar (1981 ▶); Padmavathi *et al.* (1999 ▶, 2000 ▶, 2001*a* ▶,*b* ▶). For related structures, see: Fischer *et al.* (2007*a* ▶,*b* ▶, 2008 ▶); Yao *et al.* (2006 ▶).
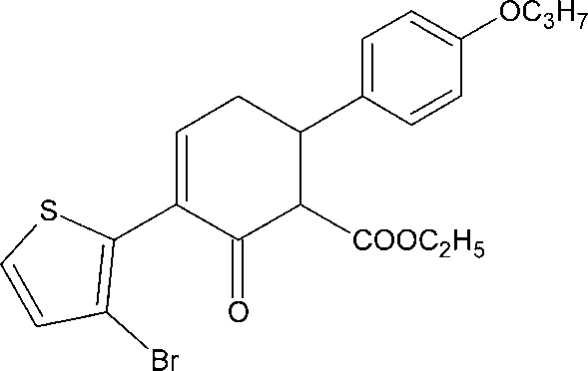


## Experimental

### 

#### Crystal data

C_22_H_23_BrO_4_S
*M* *_r_* = 463.39
Triclinic, 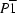

*a* = 8.809 (3) Å
*b* = 11.878 (2) Å
*c* = 20.178 (7) Å
α = 92.66 (2)°
β = 94.61 (2)°
γ = 90.16 (2)°
*V* = 2102.2 (11) Å^3^
*Z* = 4
Mo *K*α radiation
μ = 2.08 mm^−1^
*T* = 299 K
0.38 × 0.31 × 0.11 mm

#### Data collection

Bruker–Nonius KappaCCD diffractometer
Absorption correction: numerical (*HABITUS*; Herrendorf & Bärnighausen, 1997 ▶);*T*_min_ = 0.613, *T*_max_ = 0.881
31851 measured reflections
7652 independent reflections
4630 reflections with *I* > 2σ(*I*)
*R*_int_ = 0.074

#### Refinement

*R*[*F* ^2^ > 2σ(*F* ^2^)] = 0.067
*wR*(*F* ^2^) = 0.136
*S* = 1.17
7652 reflections
506 parameters
H-atom parameters constrained
Δρ_max_ = 0.45 e Å^−3^
Δρ_min_ = −0.53 e Å^−3^

### 

Data collection: *SMART* (Bruker, 1998 ▶); cell refinement: *DIRAX* (Duisenberg, 1992 ▶); data reduction: *EVALCCD* (Duisenberg *et al.*, 2003 ▶); program(s) used to solve structure: *SHELXS97* (Sheldrick, 2008 ▶); program(s) used to refine structure: *SHELXL97* (Sheldrick, 2008 ▶); molecular graphics: *DIAMOND* (Brandenburg, 1999 ▶); software used to prepare material for publication: *publCIF* (Westrip, 2008 ▶).

## Supplementary Material

Crystal structure: contains datablocks global, I. DOI: 10.1107/S1600536808032650/kp2180sup1.cif

Structure factors: contains datablocks I. DOI: 10.1107/S1600536808032650/kp2180Isup2.hkl

Additional supplementary materials:  crystallographic information; 3D view; checkCIF report

## Figures and Tables

**Fig. 1.**
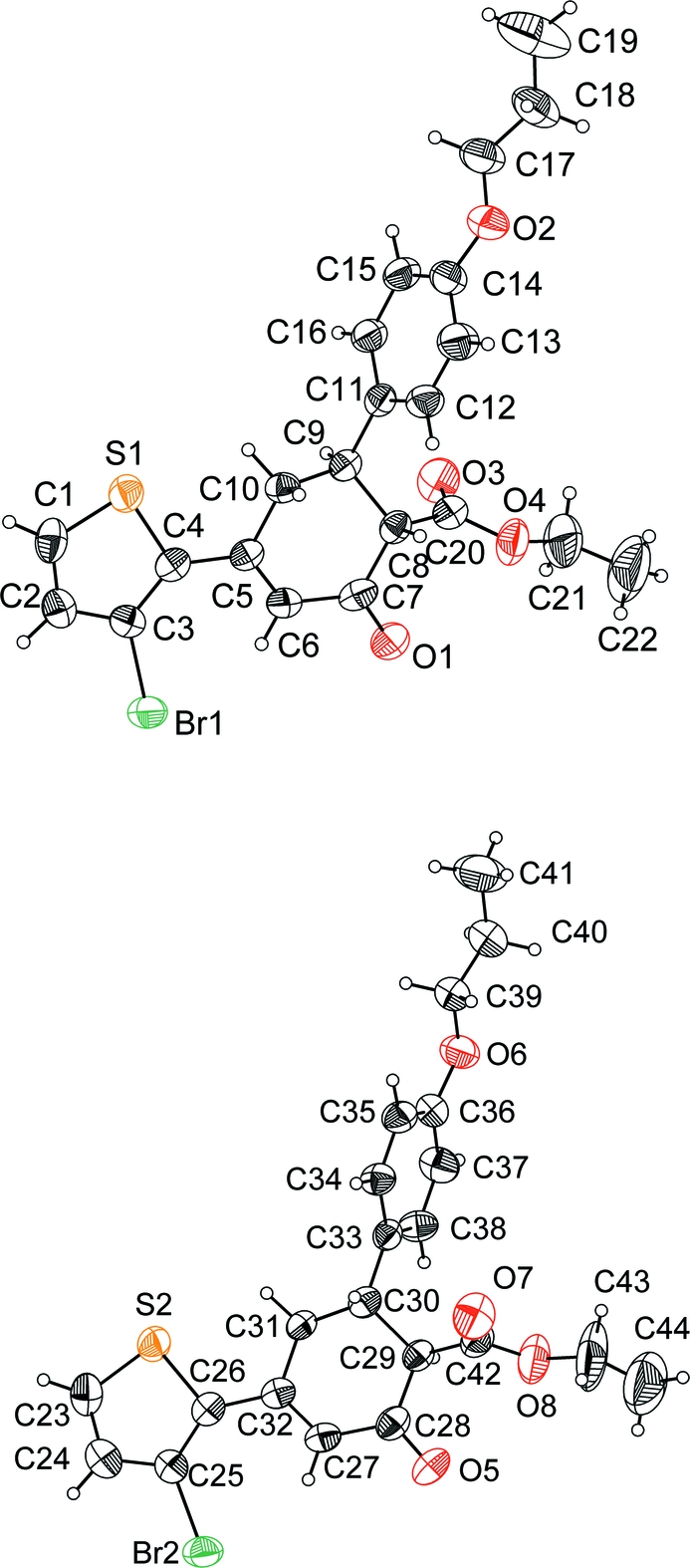
: The two molecules in the asymmetric unit. Displacement ellipsoids are drawn at the 50% probability level.

## supplementary crystallographic information

### Comment

Chalcones and the corresponding heterocyclic analogues are valuable intermediates in organic synthesis (Dhar, 1981) and exhibit a multitude of biological activities (Dimmock *et al.* 1999). From a chemical point of view, an important feature of chalcones and their heteroanalogues is the ability to act as activated unsaturated systems in conjugated addition reactions of carbanions in the presence of basic catalysts (House, 1972). This type of reaction may be exploited with the view of obtaining highly functionalized cyclohexene derivatives (Tabba *et al.*,1995) but is more commonly used for the preparation of 3,5-diaryl-6-carbethoxycyclohexanones *via* Michael addition of ethylacetoacetate. The mentioned cyclohexenones are efficient synthons in building spiranic compounds (Padmavathi *et al.*,2001) or intermediates in the synthesis of benzisoxazoles or carbazolederivatives (Padmavathi *et al.*, 1999, 2000, 2001a,b). In view of the importance of these derivatives, a new derivative *rac*-ethyl-3-(3-bromo-2-thienyl)-6-(4-propoxyphenyl)-2-oxocyclohex-3-ene-1-carboxylate, C_22_H_23_BrO_4_S was prepared and the crystal structure is reported here.

The compound is prepared by the cyclocondensation of ethyl acetoacetate with chalcone which leads to the generation of two chiral centers at C1 and C6 in the structure of cyclohexanone (I). As the reaction is not stereoselective, both configurations of the chiral carbon atoms are expected to be obtained in the synthesiszed cyclohexanone(I), which would result in a mixture of diastereomers. No attempt to separate the diastereomeric I has been undertaken and the crystals were grown from the mixture after recrystallization.

### Experimental

(2*E*)-1-(3-Bromo-2-thienyl)-3-(4-propoxyphenyl)prop-2-en-1-one(1) (1.76 g, 5 mmol) and ethyl acetoacetate (2) (0.65 g, 5 mmol) were refluxed for 2 h in 15 mL ethanol in presence of 0.8 mL 10% NaOH. The reaction mixture was cooled to room temperature and the reaction mass was filtered and recrystallized using methanol. X-ray quality crystals were grown from acetone.Yield = 67%; mp 349–351 K. CHS Calculated: 57.02, 5.00, 6.92; Observed: 56.89,4.81, 6.80.

### Refinement

Hydrogen atoms were placed at calculated positions and refined riding on the respective carrier atom. Attempts to improve the structure model using a split position for C43 and C44 resulted in an unstable refinement. Attempts to acquire data at low temperature resulted in severe deterioration of the crystal quality.

### Crystal data

**Table:**

C_22_H_23_BrO_4_S	*Z* = 4
*M**_r_* = 463.40	*F*(000) = 952
Triclinic, *P*1	*D*_x_ = 1.464 Mg m^−^^3^
Hall symbol: -P 1	Mo *K*α radiation, λ = 0.71073 Å
*a* = 8.809 (3) Å	Cell parameters from 26 reflections
*b* = 11.878 (2) Å	θ = 5.7–16.4°
*c* = 20.178 (7) Å	µ = 2.08 mm^−^^1^
α = 92.66 (2)°	*T* = 299 K
β = 94.61 (2)°	Plate, colourless
γ = 90.16 (2)°	0.38 × 0.31 × 0.11 mm
*V* = 2102.2 (11) Å^3^	

### Data collection

**Table:**

Bruker–Nonius KappaCCD diffractometer	4630 reflections with *I* > 2σ(*I*)
Radiation source: fine-focus sealed tube	*R*_int_ = 0.074
φ and ω scans	θ_max_ = 25.5°, θ_min_ = 4.6°
Absorption correction: numerical (program? reference?)	*h* = −10→10
*T*_min_ = 0.613, *T*_max_ = 0.881	*k* = −13→14
31851 measured reflections	*l* = −24→23
7652 independent reflections	

### Refinement

**Table:**

Refinement on *F*^2^	Primary atom site location: structure-invariant direct methods
Least-squares matrix: full	Secondary atom site location: difference Fourier map
*R*[*F*^2^ > 2σ(*F*^2^)] = 0.067	H-atom parameters constrained
*wR*(*F*^2^) = 0.136	*w* = 1/[σ^2^(*F*_o_^2^) + 5.8*P*] where *P* = (*F*_o_^2^ + 2*F*_c_^2^)/3
*S* = 1.17	(Δ/σ)_max_ = 0.001
7652 reflections	Δρ_max_ = 0.45 e Å^−^^3^
506 parameters	Δρ_min_ = −0.52 e Å^−^^3^
0 restraints	

### Special details

**Table:**

Geometry. All e.s.d.'s (except the e.s.d. in the dihedral angle between two l.s. planes) are estimated using the full covariance matrix. The cell e.s.d.'s are taken into account individually in the estimation of e.s.d.'s in distances, angles and torsion angles; correlations between e.s.d.'s in cell parameters are only used when they are defined by crystal symmetry. An approximate (isotropic) treatment of cell e.s.d.'s is used for estimating e.s.d.'s involving l.s. planes.
Refinement. Refinement of *F*^2^ against ALL reflections. The weighted *R*-factor *wR* and goodness of fit *S* are based on *F*^2^, conventional *R*-factors *R* are based on *F*, with *F* set to zero for negative *F*^2^. The threshold expression of *F*^2^ > σ(*F*^2^) is used only for calculating *R*-factors(gt) *etc*. and is not relevant to the choice of reflections for refinement. *R*-factors based on *F*^2^ are statistically about twice as large as those based on *F*, and *R*- factors based on ALL data will be even larger.

### Fractional atomic coordinates and isotropic or equivalent isotropic displacement parameters (Å^2^)

**Table:**

	*x*	*y*	*z*	*U*_iso_*/*U*_eq_	
Br1	1.00872 (8)	0.74502 (5)	1.07626 (3)	0.0570 (2)	
Br2	0.51193 (8)	0.76585 (5)	1.07551 (3)	0.0590 (2)	
S1	0.69191 (18)	0.46070 (13)	1.03375 (8)	0.0518 (4)	
S2	0.19207 (18)	1.04063 (14)	1.03454 (8)	0.0529 (4)	
C1	0.7253 (7)	0.4941 (5)	1.1156 (3)	0.0542 (16)	
C2	0.8199 (7)	0.5836 (5)	1.1279 (3)	0.0474 (14)	
C3	0.8687 (6)	0.6238 (4)	1.0689 (3)	0.0395 (13)	
C4	0.8093 (6)	0.5679 (4)	1.0113 (3)	0.0387 (13)	
C5	0.8252 (6)	0.5818 (4)	0.9409 (3)	0.0361 (12)	
C6	0.9291 (7)	0.6506 (4)	0.9193 (3)	0.0446 (14)	
C7	0.9453 (7)	0.6673 (4)	0.8494 (3)	0.0469 (14)	
C8	0.8412 (6)	0.6015 (4)	0.7975 (3)	0.0384 (12)	
C9	0.7790 (6)	0.4937 (4)	0.8233 (3)	0.0379 (12)	
C10	0.7154 (6)	0.5177 (4)	0.8910 (3)	0.0404 (13)	
C11	0.6635 (6)	0.4335 (4)	0.7750 (3)	0.0393 (13)	
C12	0.5401 (7)	0.4886 (5)	0.7429 (3)	0.0495 (15)	
C13	0.4415 (8)	0.4320 (5)	0.6973 (3)	0.0598 (17)	
C14	0.4589 (7)	0.3188 (5)	0.6817 (3)	0.0516 (15)	
C15	0.5740 (7)	0.2611 (5)	0.7149 (3)	0.0510 (15)	
C16	0.6750 (7)	0.3199 (4)	0.7606 (3)	0.0463 (14)	
C17	0.3718 (9)	0.1587 (6)	0.6120 (4)	0.072 (2)	
C18	0.2557 (10)	0.1339 (7)	0.5533 (4)	0.088 (3)	
C19	0.2534 (13)	0.0159 (9)	0.5288 (6)	0.144 (5)	
C20	0.9318 (7)	0.5792 (5)	0.7381 (3)	0.0495 (15)	
C21	1.0231 (10)	0.6541 (7)	0.6429 (4)	0.090 (3)	
C22	0.9822 (14)	0.7411 (8)	0.5968 (5)	0.136 (4)	
C23	0.2240 (7)	1.0186 (6)	1.1170 (3)	0.0553 (16)	
C24	0.3224 (7)	0.9319 (5)	1.1279 (3)	0.0495 (15)	
C25	0.3712 (6)	0.8839 (4)	1.0692 (3)	0.0399 (13)	
C26	0.3119 (6)	0.9313 (4)	1.0117 (3)	0.0376 (12)	
C27	0.4301 (6)	0.8377 (4)	0.9187 (3)	0.0450 (14)	
C28	0.4458 (7)	0.8132 (4)	0.8486 (3)	0.0478 (14)	
C29	0.3417 (6)	0.8698 (4)	0.7973 (3)	0.0399 (13)	
C30	0.2803 (6)	0.9815 (4)	0.8246 (3)	0.0396 (13)	
C31	0.2186 (6)	0.9668 (4)	0.8918 (3)	0.0412 (13)	
C32	0.3274 (6)	0.9096 (4)	0.9413 (3)	0.0386 (13)	
C33	0.1648 (6)	1.0349 (4)	0.7757 (3)	0.0395 (13)	
C34	0.1797 (7)	1.1466 (4)	0.7618 (3)	0.0457 (14)	
C35	0.0795 (7)	1.1981 (5)	0.7162 (3)	0.0513 (15)	
C36	−0.0374 (7)	1.1362 (5)	0.6834 (3)	0.0477 (14)	
C37	−0.0588 (7)	1.0261 (5)	0.6991 (3)	0.0561 (16)	
C38	0.0409 (7)	0.9759 (5)	0.7438 (3)	0.0505 (15)	
C39	−0.1223 (8)	1.2903 (5)	0.6170 (3)	0.0592 (17)	
C40	−0.2396 (9)	1.3122 (6)	0.5615 (4)	0.073 (2)	
C41	−0.2367 (12)	1.4334 (7)	0.5416 (5)	0.112 (3)	
C42	0.4294 (7)	0.8853 (5)	0.7374 (3)	0.0518 (15)	
C43	0.5140 (15)	0.7996 (8)	0.6391 (5)	0.142 (5)	
C44	0.5026 (15)	0.7049 (9)	0.6013 (5)	0.146 (5)	
O1	1.0389 (6)	0.7333 (4)	0.8325 (2)	0.0744 (14)	
O2	0.3581 (5)	0.2734 (3)	0.6319 (2)	0.0654 (12)	
O3	1.0136 (5)	0.5001 (4)	0.7321 (2)	0.0650 (12)	
O4	0.9151 (6)	0.6605 (4)	0.6946 (2)	0.0655 (12)	
O5	0.5407 (6)	0.7450 (4)	0.8317 (2)	0.0753 (14)	
O6	−0.1381 (5)	1.1766 (3)	0.6339 (2)	0.0608 (12)	
O7	0.5113 (6)	0.9633 (4)	0.7307 (2)	0.0740 (14)	
O8	0.4101 (7)	0.7991 (4)	0.6931 (2)	0.0820 (16)	
H1	0.6832	0.4552	1.1487	0.065*	
H2	0.8495	0.6145	1.1702	0.057*	
H6	0.9947	0.6899	0.9506	0.054*	
H8	0.7551	0.6497	0.7841	0.046*	
H9	0.8651	0.4425	0.8306	0.046*	
H10A	0.6225	0.5609	0.8845	0.048*	
H10B	0.6893	0.4466	0.9092	0.048*	
H12	0.5251	0.5648	0.7526	0.059*	
H13	0.3610	0.4706	0.6764	0.072*	
H15	0.5843	0.1838	0.7071	0.061*	
H16	0.7535	0.2804	0.7825	0.056*	
H17A	0.3522	0.1114	0.6484	0.087*	
H17B	0.4738	0.1435	0.5992	0.087*	
H18A	0.1552	0.1540	0.5663	0.105*	
H18B	0.2782	0.1810	0.5173	0.105*	
H19A	0.3530	−0.0052	0.5167	0.173*	
H19B	0.1815	0.0064	0.4906	0.173*	
H19C	0.2243	−0.0310	0.5632	0.173*	
H21A	1.1263	0.6663	0.6625	0.108*	
H21B	1.0175	0.5805	0.6199	0.108*	
H22A	0.8867	0.7220	0.5726	0.163*	
H22B	1.0596	0.7469	0.5662	0.163*	
H22C	0.9733	0.8120	0.6210	0.163*	
H23	0.1803	1.0609	1.1504	0.066*	
H24	0.3530	0.9072	1.1699	0.059*	
H27	0.4951	0.8018	0.9494	0.054*	
H29	0.2553	0.8193	0.7840	0.048*	
H30	0.3669	1.0337	0.8320	0.048*	
H31A	0.1253	0.9228	0.8851	0.049*	
H31B	0.1930	1.0404	0.9105	0.049*	
H34	0.2591	1.1888	0.7837	0.055*	
H35	0.0921	1.2737	0.7077	0.062*	
H37	−0.1420	0.9855	0.6791	0.067*	
H38	0.0258	0.9009	0.7531	0.061*	
H39A	−0.0211	1.3035	0.6032	0.071*	
H39B	−0.1374	1.3404	0.6553	0.071*	
H40A	−0.3397	1.2947	0.5751	0.088*	
H40B	−0.2217	1.2628	0.5233	0.088*	
H41A	−0.2348	1.4829	0.5806	0.135*	
H41B	−0.3260	1.4480	0.5128	0.135*	
H41C	−0.1475	1.4462	0.5185	0.135*	
H43A	0.4900	0.8631	0.6117	0.170*	
H43B	0.6181	0.8089	0.6583	0.170*	
H44A	0.5363	0.6427	0.6273	0.218*	
H44B	0.5648	0.7106	0.5646	0.218*	
H44C	0.3984	0.6928	0.5846	0.218*	

### Atomic displacement parameters (Å^2^)

**Table:**

	*U*^11^	*U*^22^	*U*^33^	*U*^12^	*U*^13^	*U*^23^
Br1	0.0682 (5)	0.0402 (3)	0.0602 (4)	−0.0072 (3)	−0.0073 (3)	−0.0002 (3)
Br2	0.0684 (5)	0.0400 (3)	0.0662 (5)	0.0054 (3)	−0.0103 (3)	0.0053 (3)
S1	0.0468 (9)	0.0620 (9)	0.0474 (9)	−0.0140 (7)	0.0051 (7)	0.0089 (7)
S2	0.0447 (9)	0.0639 (10)	0.0497 (10)	0.0139 (7)	0.0043 (7)	−0.0032 (7)
C1	0.047 (4)	0.072 (4)	0.046 (4)	0.001 (3)	0.010 (3)	0.018 (3)
C2	0.045 (4)	0.054 (3)	0.043 (4)	0.010 (3)	0.002 (3)	0.002 (3)
C3	0.035 (3)	0.036 (3)	0.047 (3)	0.009 (2)	−0.003 (3)	−0.002 (2)
C4	0.034 (3)	0.035 (3)	0.049 (4)	0.007 (2)	0.007 (3)	0.004 (2)
C5	0.033 (3)	0.030 (3)	0.046 (3)	0.007 (2)	0.004 (2)	0.004 (2)
C6	0.049 (4)	0.038 (3)	0.046 (4)	−0.008 (3)	0.003 (3)	−0.001 (3)
C7	0.048 (4)	0.032 (3)	0.061 (4)	−0.002 (3)	0.013 (3)	0.003 (3)
C8	0.040 (3)	0.036 (3)	0.041 (3)	0.002 (2)	0.010 (2)	0.005 (2)
C9	0.036 (3)	0.035 (3)	0.043 (3)	−0.001 (2)	0.003 (2)	0.006 (2)
C10	0.038 (3)	0.038 (3)	0.046 (3)	−0.006 (2)	0.005 (3)	0.000 (2)
C11	0.042 (3)	0.037 (3)	0.040 (3)	0.000 (2)	0.007 (3)	0.005 (2)
C12	0.056 (4)	0.037 (3)	0.055 (4)	0.002 (3)	0.002 (3)	0.000 (3)
C13	0.057 (4)	0.052 (4)	0.068 (5)	0.000 (3)	−0.010 (3)	0.004 (3)
C14	0.056 (4)	0.052 (4)	0.046 (4)	−0.009 (3)	0.004 (3)	−0.005 (3)
C15	0.063 (4)	0.035 (3)	0.056 (4)	−0.001 (3)	0.008 (3)	0.003 (3)
C16	0.048 (4)	0.039 (3)	0.052 (4)	−0.002 (3)	0.001 (3)	0.005 (3)
C17	0.085 (5)	0.066 (4)	0.065 (5)	−0.022 (4)	0.005 (4)	−0.015 (4)
C18	0.100 (6)	0.093 (6)	0.065 (5)	−0.023 (5)	−0.009 (4)	−0.021 (4)
C19	0.134 (10)	0.129 (9)	0.156 (11)	−0.018 (7)	−0.031 (8)	−0.064 (8)
C20	0.057 (4)	0.044 (3)	0.048 (4)	−0.008 (3)	0.005 (3)	0.004 (3)
C21	0.113 (7)	0.106 (6)	0.057 (5)	−0.006 (5)	0.039 (5)	0.012 (4)
C22	0.208 (13)	0.123 (8)	0.088 (7)	0.016 (8)	0.070 (8)	0.040 (6)
C23	0.040 (4)	0.075 (4)	0.049 (4)	−0.004 (3)	0.005 (3)	−0.012 (3)
C24	0.040 (3)	0.059 (4)	0.049 (4)	−0.019 (3)	−0.003 (3)	0.004 (3)
C25	0.039 (3)	0.036 (3)	0.044 (3)	−0.015 (2)	−0.003 (3)	0.001 (2)
C26	0.024 (3)	0.038 (3)	0.051 (4)	−0.009 (2)	0.001 (2)	−0.004 (2)
C27	0.039 (3)	0.039 (3)	0.056 (4)	0.005 (3)	0.000 (3)	0.004 (3)
C28	0.051 (4)	0.036 (3)	0.057 (4)	−0.001 (3)	0.008 (3)	−0.003 (3)
C29	0.039 (3)	0.034 (3)	0.047 (3)	−0.001 (2)	0.007 (3)	−0.001 (2)
C30	0.037 (3)	0.033 (3)	0.049 (3)	−0.004 (2)	0.003 (3)	−0.001 (2)
C31	0.044 (3)	0.035 (3)	0.044 (3)	0.008 (2)	0.004 (3)	−0.002 (2)
C32	0.033 (3)	0.030 (3)	0.051 (4)	−0.008 (2)	0.001 (3)	0.001 (2)
C33	0.044 (3)	0.034 (3)	0.041 (3)	0.000 (2)	0.009 (3)	0.000 (2)
C34	0.047 (4)	0.036 (3)	0.053 (4)	−0.004 (3)	−0.003 (3)	0.001 (3)
C35	0.061 (4)	0.036 (3)	0.056 (4)	0.001 (3)	0.002 (3)	0.003 (3)
C36	0.052 (4)	0.050 (3)	0.041 (3)	0.003 (3)	0.002 (3)	0.004 (3)
C37	0.056 (4)	0.051 (4)	0.059 (4)	−0.012 (3)	−0.012 (3)	0.005 (3)
C38	0.050 (4)	0.036 (3)	0.066 (4)	−0.003 (3)	0.002 (3)	0.010 (3)
C39	0.069 (5)	0.061 (4)	0.048 (4)	0.013 (3)	0.003 (3)	0.008 (3)
C40	0.089 (6)	0.076 (5)	0.055 (4)	0.015 (4)	0.000 (4)	0.011 (4)
C41	0.146 (9)	0.085 (6)	0.101 (7)	0.021 (6)	−0.030 (6)	0.025 (5)
C42	0.055 (4)	0.047 (3)	0.055 (4)	0.009 (3)	0.014 (3)	−0.001 (3)
C43	0.253 (14)	0.097 (7)	0.087 (7)	−0.033 (8)	0.105 (8)	−0.031 (6)
C44	0.207 (13)	0.126 (9)	0.114 (9)	0.013 (9)	0.083 (9)	−0.009 (7)
O1	0.094 (4)	0.073 (3)	0.058 (3)	−0.044 (3)	0.013 (3)	0.008 (2)
O2	0.075 (3)	0.056 (3)	0.062 (3)	−0.015 (2)	−0.014 (2)	−0.003 (2)
O3	0.073 (3)	0.059 (3)	0.066 (3)	0.016 (2)	0.024 (2)	0.002 (2)
O4	0.088 (4)	0.065 (3)	0.048 (3)	0.008 (2)	0.022 (2)	0.018 (2)
O5	0.087 (4)	0.066 (3)	0.075 (3)	0.037 (3)	0.017 (3)	−0.006 (2)
O6	0.071 (3)	0.053 (2)	0.057 (3)	0.003 (2)	−0.010 (2)	0.009 (2)
O7	0.082 (4)	0.063 (3)	0.082 (4)	−0.019 (3)	0.034 (3)	0.006 (2)
O8	0.125 (5)	0.062 (3)	0.062 (3)	−0.008 (3)	0.038 (3)	−0.014 (2)

### Geometric parameters (Å, °)

**Table:**

Br1—C3	1.887 (5)	C39—O6	1.418 (7)
Br2—C25	1.877 (6)	C39—C40	1.495 (9)
S1—C1	1.682 (6)	C40—C41	1.513 (10)
S1—C4	1.737 (5)	C42—O7	1.192 (7)
S2—C23	1.697 (7)	C42—O8	1.329 (7)
S2—C26	1.744 (5)	C43—C44	1.329 (12)
C1—C2	1.350 (8)	C43—O8	1.480 (9)
C2—C3	1.402 (8)	C1—H1	0.9300
C3—C4	1.375 (7)	C2—H2	0.9301
C4—C5	1.456 (7)	C6—H6	0.9300
C5—C6	1.338 (7)	C8—H8	0.9800
C5—C10	1.516 (7)	C9—H9	0.9800
C6—C7	1.451 (8)	C10—H10A	0.9700
C7—O1	1.216 (6)	C10—H10B	0.9700
C7—C8	1.521 (8)	C12—H12	0.9299
C8—C20	1.507 (8)	C13—H13	0.9301
C8—C9	1.522 (7)	C15—H15	0.9300
C9—C11	1.506 (7)	C16—H16	0.9300
C9—C10	1.533 (7)	C17—H17A	0.9699
C11—C16	1.372 (7)	C17—H17B	0.9700
C11—C12	1.400 (8)	C18—H18A	0.9700
C12—C13	1.364 (8)	C18—H18B	0.9700
C13—C14	1.378 (8)	C19—H19A	0.9600
C14—C15	1.373 (8)	C19—H19B	0.9600
C14—O2	1.376 (7)	C19—H19C	0.9600
C15—C16	1.391 (8)	C21—H21A	0.9700
C17—O2	1.411 (7)	C21—H21B	0.9700
C17—C18	1.518 (9)	C22—H22A	0.9600
C18—C19	1.464 (11)	C22—H22B	0.9599
C20—O3	1.193 (7)	C22—H22C	0.9601
C20—O4	1.335 (7)	C23—H23	0.9300
C21—C22	1.449 (11)	C24—H24	0.9299
C21—O4	1.467 (8)	C27—H27	0.9301
C23—C24	1.363 (8)	C29—H29	0.9800
C24—C25	1.390 (8)	C30—H30	0.9800
C25—C26	1.378 (7)	C31—H31A	0.9700
C26—C32	1.450 (7)	C31—H31B	0.9700
C27—C32	1.338 (7)	C34—H34	0.9300
C27—C28	1.448 (8)	C35—H35	0.9299
C28—O5	1.223 (7)	C37—H37	0.9300
C28—C29	1.510 (8)	C38—H38	0.9300
C29—C42	1.503 (8)	C39—H39A	0.9700
C29—C30	1.530 (7)	C39—H39B	0.9701
C30—C31	1.519 (7)	C40—H40A	0.9700
C30—C33	1.519 (7)	C40—H40B	0.9700
C31—C32	1.514 (7)	C41—H41A	0.9600
C33—C34	1.376 (7)	C41—H41B	0.9601
C33—C38	1.393 (8)	C41—H41C	0.9600
C34—C35	1.386 (8)	C43—H43A	0.9700
C35—C36	1.371 (8)	C43—H43B	0.9700
C36—C37	1.376 (8)	C44—H44A	0.9600
C36—O6	1.385 (7)	C44—H44B	0.9600
C37—C38	1.366 (8)	C44—H44C	0.9600
			
C1—S1—C4	93.0 (3)	C10—C9—H9	107.0
C23—S2—C26	93.2 (3)	C5—C10—H10A	108.8
C2—C1—S1	112.6 (5)	C9—C10—H10A	108.8
C1—C2—C3	111.4 (5)	C5—C10—H10B	108.9
C4—C3—C2	115.2 (5)	C9—C10—H10B	108.8
C4—C3—Br1	127.1 (4)	H10A—C10—H10B	107.7
C2—C3—Br1	117.6 (4)	C13—C12—H12	119.4
C3—C4—C5	133.8 (5)	C11—C12—H12	119.6
C3—C4—S1	107.7 (4)	C12—C13—H13	119.3
C5—C4—S1	118.5 (4)	C14—C13—H13	119.3
C6—C5—C4	122.6 (5)	C14—C15—H15	120.6
C6—C5—C10	119.6 (5)	C16—C15—H15	120.4
C4—C5—C10	117.8 (5)	C11—C16—H16	118.6
C5—C6—C7	123.6 (5)	C15—C16—H16	118.5
O1—C7—C6	120.9 (5)	O2—C17—H17A	110.1
O1—C7—C8	120.4 (5)	C18—C17—H17A	110.1
C6—C7—C8	118.7 (5)	O2—C17—H17B	110.2
C20—C8—C7	106.6 (5)	C18—C17—H17B	110.3
C20—C8—C9	112.3 (4)	H17A—C17—H17B	108.5
C7—C8—C9	112.9 (4)	C19—C18—H18A	109.0
C11—C9—C8	113.7 (4)	C17—C18—H18A	108.9
C11—C9—C10	111.4 (4)	C19—C18—H18B	108.7
C8—C9—C10	110.3 (4)	C17—C18—H18B	108.8
C5—C10—C9	113.6 (4)	H18A—C18—H18B	107.7
C16—C11—C12	116.6 (5)	C18—C19—H19A	109.6
C16—C11—C9	120.7 (5)	C18—C19—H19B	109.5
C12—C11—C9	122.7 (5)	H19A—C19—H19B	109.5
C13—C12—C11	120.9 (5)	C18—C19—H19C	109.3
C12—C13—C14	121.4 (6)	H19A—C19—H19C	109.5
C15—C14—O2	125.2 (5)	H19B—C19—H19C	109.5
C15—C14—C13	119.1 (6)	C22—C21—H21A	110.1
O2—C14—C13	115.7 (6)	O4—C21—H21A	110.3
C14—C15—C16	119.0 (5)	C22—C21—H21B	110.3
C11—C16—C15	122.9 (5)	O4—C21—H21B	110.2
O2—C17—C18	107.6 (6)	H21A—C21—H21B	108.5
C19—C18—C17	113.6 (8)	C21—C22—H22A	109.4
O3—C20—O4	124.1 (6)	C21—C22—H22B	109.5
O3—C20—C8	123.6 (5)	H22A—C22—H22B	109.5
O4—C20—C8	112.3 (5)	C21—C22—H22C	109.5
C22—C21—O4	107.4 (7)	H22A—C22—H22C	109.5
C24—C23—S2	111.3 (5)	H22B—C22—H22C	109.5
C23—C24—C25	112.6 (6)	C24—C23—H23	124.5
C26—C25—C24	115.3 (5)	S2—C23—H23	124.2
C26—C25—Br2	126.7 (4)	C23—C24—H24	123.7
C24—C25—Br2	118.0 (4)	C25—C24—H24	123.7
C25—C26—C32	134.8 (5)	C32—C27—H27	118.4
C25—C26—S2	107.7 (4)	C28—C27—H27	118.2
C32—C26—S2	117.5 (4)	C42—C29—H29	108.3
C32—C27—C28	123.4 (5)	C28—C29—H29	108.4
O5—C28—C27	119.7 (6)	C30—C29—H29	108.5
O5—C28—C29	120.8 (6)	C31—C30—H30	106.9
C27—C28—C29	119.5 (5)	C33—C30—H30	106.9
C42—C29—C28	107.6 (5)	C29—C30—H30	107.0
C42—C29—C30	111.9 (4)	C32—C31—H31A	108.7
C28—C29—C30	112.0 (5)	C30—C31—H31A	108.6
C31—C30—C33	112.3 (4)	C32—C31—H31B	108.9
C31—C30—C29	110.5 (4)	C30—C31—H31B	108.7
C33—C30—C29	112.8 (4)	H31A—C31—H31B	107.6
C32—C31—C30	114.2 (5)	C33—C34—H34	119.0
C27—C32—C26	122.5 (5)	C35—C34—H34	119.0
C27—C32—C31	119.0 (5)	C36—C35—H35	120.3
C26—C32—C31	118.5 (5)	C34—C35—H35	120.3
C34—C33—C38	117.2 (5)	C38—C37—H37	119.7
C34—C33—C30	120.0 (5)	C36—C37—H37	119.7
C38—C33—C30	122.8 (5)	C37—C38—H38	119.4
C33—C34—C35	122.0 (5)	C33—C38—H38	119.4
C36—C35—C34	119.4 (5)	O6—C39—H39A	110.2
C35—C36—C37	119.5 (6)	C40—C39—H39A	110.2
C35—C36—O6	124.4 (5)	O6—C39—H39B	110.0
C37—C36—O6	116.1 (5)	C40—C39—H39B	110.1
C38—C37—C36	120.7 (6)	H39A—C39—H39B	108.5
C37—C38—C33	121.2 (5)	C39—C40—H40A	109.2
O6—C39—C40	107.9 (5)	C41—C40—H40A	109.1
C39—C40—C41	112.1 (7)	C39—C40—H40B	109.1
O7—C42—O8	123.2 (6)	C41—C40—H40B	109.3
O7—C42—C29	124.7 (6)	H40A—C40—H40B	107.9
O8—C42—C29	112.1 (5)	C40—C41—H41A	109.6
C44—C43—O8	111.6 (9)	C40—C41—H41B	109.4
C14—O2—C17	118.9 (5)	H41A—C41—H41B	109.5
C20—O4—C21	114.1 (5)	C40—C41—H41C	109.4
C36—O6—C39	118.1 (5)	H41A—C41—H41C	109.5
C42—O8—C43	114.5 (6)	H41B—C41—H41C	109.5
C2—C1—H1	123.7	C44—C43—H43A	109.3
S1—C1—H1	123.7	O8—C43—H43A	109.3
C1—C2—H2	124.4	C44—C43—H43B	109.3
C3—C2—H2	124.2	O8—C43—H43B	109.3
C5—C6—H6	118.3	H43A—C43—H43B	108.0
C7—C6—H6	118.1	C43—C44—H44A	109.5
C20—C8—H8	108.2	C43—C44—H44B	109.5
C7—C8—H8	108.2	H44A—C44—H44B	109.5
C9—C8—H8	108.4	C43—C44—H44C	109.5
C11—C9—H9	107.0	H44A—C44—H44C	109.5
C8—C9—H9	107.1	H44B—C44—H44C	109.5
